# Editorial: Sexual behavior research: towards an understanding of CNS and spinal cord modulation of male sexual behavior and sexual dysfunctions

**DOI:** 10.3389/fnins.2024.1422477

**Published:** 2024-05-10

**Authors:** Gabriel Gutiérrez-Ospina, Michal Bialy

**Affiliations:** ^1^Departmento de Biología Celular y Fisiología, Instituto de Investigaciones Biomédicas, Universidad Nacional Autónoma de México, Ciudad de México, México; ^2^Department of Experimental and Clinical Physiology, Medical University of Warsaw, Warsaw, Poland

**Keywords:** sexual motivation, sexual reward, premature ejaculation, sexual arousal, endocannabinoids, selective serotonin reuptake inhibitors, brain sexual circuits

The study of the processes and mechanisms underlying male sexual behavior in mammals is an attractive scientific endeavor not only for its intrinsic ecological/evolutionary importance, but also for its implications for understanding human sexual life, health, and dysfunction. Although decades of research have identified the “archetypal” neurocircuits and neurochemical-endocrine loops that ensure successful male sexual behavior in a variety of wild and domesticated mammals, there are numerous ongoing issues at various levels of biological organization that require further investigation. Three of the articles submitted to this Research Topic address the regulation of the functional morphology of male sexual behavior.

Gaytán-Tocavén et al. provided functional morphological evidence suggesting that the olfactory bulbs, bed nucleus of the *stria terminalis*, amygdala, medial preoptic area, ventromedial hypothalamus, ventral tegmental area, nucleus *accumbens*, and neocortex modulate male sexual motivation as a function of prior previous sexual experience. This was done by using a new technique that combines magnetic resonance imaging and the administration of MnCl_2_ (i.e., manganese-enhanced magnetic resonance imaging), a procedure that allows brain activity to be analyzed after rats are allowed to have sexual intercourse.

Rodríguez-Manzo and Canseco-Alba explored the neurochemical basis of male sexual activity. These authors convincingly argued that it is possible to restore male sexual motivation in sexually satiated male rats after antagonizing the endocannabinoid (CB) transmission in the mesolimbic dopaminergic system. Moreover, blockade of CB receptors in the ventral tegmental area during copulation to satiation disrupts the long-lasting inhibition of sexual motivation. Their reflections and work may help to understand the role of the endocannabinoid system in hypoactive sexual desire disorder and plasticity mechanisms in the motivation/reward system.

Olivier et al. tested whether the chronic use of selective serotonin reuptake inhibitors (SSRIs) would delay ejaculation in rats with relatively short ejaculatory latencies, a condition somewhat reminiscent of premature ejaculation in men. As expected, the combined administration of SSRIs and the 5HT1A receptor antagonist (Atlas987) increased, in a dose-dependent manner, ejaculatory latency in rats so treated. These results open the possibility of providing some relief to patients who complain of prolonged premature ejaculation in the near future.

Arousal and motivation are intertwined physiological processes that define the intensity of the output of male sexual behavior. Although each term has an intuitive definition, researchers continue to debate its conceptual meaning, the best methods for quantifying it, and the functional anatomical substrate that underlies it. Two papers in this volume address these important issues:

Ventura-Aquino and Ågmo presented their view on sexual motivation, a biological process that is difficult to define and analyze objectively. In their work, they suggested using the notion of a central motive state instead of motivation, as it may have a more materialistic meaning, thus providing researchers with directions to find its neurobiological correlate and substrate while uncovering better ways to quantify it. Ventura-Aquino and Ågmo speculated that the central motive state could be analyzed by studying the electrophysiological responses of neurons located at sites in the central and peripheral nervous systems where responsiveness to sexual stimuli is thought to be organized.

Bogacki-Rychlik et al. undertook a similar quest but in the context of male sexual arousal. They suggested the existence of separate states of general arousal and sexual arousal, emergent processes involving several autonomic/somatic neural systems themselves interconnected through general and local functional loops that prepare males to engage in sexual behavior before they are motivated to do so. In this context, male sexual arousal would be prompted by a set of autonomic reactions elicited by sensory inputs that are analyzed at different brain levels, all leading to the occurrence of penile erection and potentially to ejaculation. Their definition emphasizes the importance of interoceptive processes leading to male sexual arousal and provides a conceptual framework that allows scientists and clinicians to analyze arousal by detecting autonomic parameters while separating them from motivational components.

Ventura-Aquino and Ågmo and Bogacki-Rychlik et al. emphasized the importance of defining an adequate set of variables to precisely parameterize sexual behavior, motivation, and arousal. This is a fundamental step in translating conceptual models, which are largely supported by animal experimental data, to the human clinical setting. Is it better to reduce or increase the number of variables to parameterize sexual behavior? While some authors believe that looking for “single golden parameters” in female sexual behavior is the way to go (e.g., Pfaff, 2017), especially in male sexual activity others argue it is better to identify more (Sachs, 2000, 2008; Trejo-Sánchez et al., 2020). How the search for a unique “golden parameter” can lead astray is indicated by the reviews presented in the series co-authored by Ventura-Aquino and Ågmo (Le Moëne and Ågmo, 2019). Thus, it seems that, with the right approach, addressing the diversity of processes that regulate male behavior can provide a platform for understanding and translating animal research into the pathophysiology of sexual behavior in humans as well (Bialy et al., 2019).

Ultimately, what the academic editors of “*Sexual behavior research: toward an understanding of central nervous system and spinal cord modulation of male sexual behavior and sexual dysfunctions*” hoped was to incite a productive conversation about topics that could help advance existing knowledge about the neurobiological foundations of male sexual behavior, and the implications that this might have for human clinical practice. We hope that the reader will find our efforts valuable.

## Author contributions

GG-O: Conceptualization, Writing – original draft, Writing – review & editing. MB: Conceptualization, Writing – original draft, Writing – review & editing.
